# ^13^C-NMR Data of Three Important Diterpenes Isolated from *Euphorbia* Species

**DOI:** 10.3390/molecules14114454

**Published:** 2009-11-06

**Authors:** Qi-Cheng Wu, Yu-Ping Tang, An-Wei Ding, Fen-Qiang You, Li Zhang, Jin-Ao Duan

**Affiliations:** 1 Jiangsu Key Laboratory for TCM Formulae Research, Nanjing University of Chinese Medicine, Nanjing 210046, China; 2 Affiliated Hospital, Nanjing University of Chinese Medicine, Kunshan, China

**Keywords:** *Euphorbia*, diterpene, abietane, tigliane, ingenane, ^13^C-NMR data

## Abstract

*Euphorbia* species are widely distributed plants, many of which are used in folk medicine. Over the past twenty years, they have received considerable phytochemical and biological attention. Their diterpenoid constituents, especially those with abietane, tigliane, ingenane skeletons, are thought to be the main toxicant and bioactive factors. In this work, the utility of ^13^C-NMR spectroscopy for the structural elucidation of these compounds is briefly discussed.

## 1. Introduction

The *Euphorbia* is the largest genus in the plant family Euphorbiaceae, comprising about 2,000 known species [1]. *Euphorbia* are widely distributed throughout both hemispheres and range in morphology from large desert succulents to trees and even some small herbaceous plant types. Researched parts in various *Euphorbia* species include the roots, seeds, latex, lactiferous tubes, stem wood, stem barks, leaves, and whole plants.

Many studies have suggested that these plants have not only therapeutic relevance but that they also display toxicity [2]. Some constituents of *Euphorbia* species may be promising lead compounds for drug development. Certain *Euphorbia* species have been reported to possess antitumor activity and have been recommended for use as anticancer remedies [3,4]. Their antitumor activity was mainly attributed to the presence of abietane diterpene derivatives, most of which contain lactone structures reported to possess potent antineoplastic activity toards various cancer cell lines [5,6,7,8,9]. Moreover, some *Euphorbia* species have been also used as medicinal plants for the treatment of skin diseases, gonorrhea, migraines, intestinal parasites, warts and for mediating pain perception [10,11,12]. Many researchers have shown that *Euphorbia* species also possess antiproliferative activity [13], cytotoxicity [14], antimicrobial activity [15], antipyretic-analgesic activity [16], inhibition of HIV-1 viral infection [17], inhibitory activity on the mammalian mitochondrial respiratory chain [18], etc.

As mentioned, there are also some reports of toxicity in *Euphorbia* species. Their toxic substances originate from the milky sap, which is a deterrent to insects and herbivores [19]. Besides, they may possess extreme proinflammatory and tumor promoting toxicities [20,21]. Severe pain and inflammation can result from contact with the eyes, nose, mouth and even skin, which may be due to the activation of protein kinase C enzyme [22]. The toxic constituents of *Euphorbia* species were considered to be a kind of specific diterpenes, globally called phorboids, which comprise tigliane, ingenane and daphnane diterpene derivatives [23,24].

Terpenes, including diterpenes and triterpenes, have been frequently found in *Euphorbia* species. Steroids, cerebrosides, glycerols, phenolics and flavonoids were also isolated from plants of the genus [10], but the compounds most relevant to the toxicity and considerable biological activities in *Euphorbia* are diterpenes, especially those with abietane, tigliane, and ingenane skeletons [10].

Many researchers have suggested that there was a close relationship between the structures and the biological activity, so the structure elucidation is very important for these diterpenes. In this review article, we summarize the ^13^C-NMR data of these three important diterpene skeleton types of *Euphorbia* species, covering 42 abietanes, 51 ingenanes and 30 tiglianes. The structure-activity relationship and the features on the chemical shifts were also briefly discussed.

## 2. Abietane Derivates Isolated from *Euphorbia* Species (Table 1)

Most plants of the genus *Euphorbia* contain abietane diterpenoids, which usually have an extra α,β-unsaturated γ-lactone ring located between C-12 and C-13, and some of which have an epoxy ring at C-8 and C-14, or C-11 and C-12, as is the case of **7**–**14**. Some carbons of these diterpenes, especially C-8, C-14, C-11 and C-12 are frequently substituted by hydroxyl groups or form double bonds. Compounds **32**–**36** indicate that the 18-Me and C-3 could form a three-membered ring. In addition, some abietanes (**39**–**42**) without lactone rings were also isolated from the genus *Euphorbia.* Many abietane diterpenoids exhibit inhibitive activity on various types of tumor cells, such as ANA-1, B16, Jurkat cells [25], K562 cells [7] and LNCaP cells [6]. By comparing the active compound **8** with the inactive one **12**, it could be concluded that the C-11/C-12 epoxy ring system was necessary in mediating cytotoxicity. Compounds **2** and **3** are diastereomers, differing only in the stereochemistry at the chiral centers C-8 and C-14, but only compound **2** showed activity, which suggested that the ring C configuration is also crucial for the activity [25]. The α,β-unsaturated lactone is not the only necessary group for the cytotoxic effects, since compounds **3** and **12** do not show cytotoxicity [25]. In addition, the similar compounds **18**, **19**, **26** and **28** were tested in the inhibition of P-glycoprotein transport activity. The higher inhibitory effect of **26** might be derived from the carbonyl position at C-2, most probably due to the conformational and functional changes in the P-gp induced by the particular structures of helioscopinolides [26].

**Table 1:** Abietane diterpenoids isolated from *Euphorbia* species.

No	Name	Species	Ref
**1**	7β-Hydroxy-8α,14-dihydro jolkinolide E	*E. terracina*	[27]
**2**	Yuexiandajisu D	*E. ebracteolata*	[25]
**3**	Yuexiandajisu E	*E. ebracteolata*	[25]
**4**	*ent*-8β,14α-Dihydroxy-13(15)-ene-16(12β)-abietanolide	*E. wallichii*	[28]
**5**	*ent*-8β,14β-Dihydroxy-13(15)-ene-16(12β)-abietanolide	*E. wallichii*	[28]
**6**	Ebracteolatanolide B	*E. ebracteolata*	[25]
**7**	Ebracteolatanolide A	*E. ebracteolata*	[25]
**8**	Jolkinolide B	*E. fischeriana,*	[29]
		*E. sessiliflora*	[30]
**9**	17-Hydroxyjolkinolide B	*E. fischeriana*	[31]
**10**	17-Acetoxyjolkinolide B	*E. fischeriana*	[31]
**11**	17-Acetoxyjolkinolide A	*E. fischeriana*	[32]
**12**	Jolkinolide A	*E. wallichii*	[28]
		*E. fischeriana*	[29]
		*E. fidjiana*	[33]
		*E. guyoniana*	[34]
**13**	17-Hydroxyjolkinolide A	*E. fischeriana*	[32]
		*E. fidjiana*	[33]
**14**	3α-Hydroxyjolkinolide A	*E. wallichii*	[28]
**15**	7β-Hydroxy-*ent*-abieta-8(14),13(15)-dien-12α,16-olide	*E. seguieriana*	[35]
**16**	7β,9β-Dihydroxy-*ent*-abieta-8(14),13(15)-dien-12α,16-olide	*E. seguieriana*	[35]
**17**	*ent*-Abieta-8(14),13(15)-dien-16,12-olide [Jolkinolide E]	*E. fidjiana*	[33]
		*E. characias*	[34]
		*E. guyoniana*	[36]
**18**	Helioscopinolide A	*E. pubescens*	[37]
		*E. semiperfoliata*	[38]
		*E. helioscopia*	[39]
**19**	Helioscopinolide B	*E. pubescens*	[37]
		*E. semiperfoliata*	[38]
		*E. helioscopia*	[39]
		*E. calyptrata*	[40]
**20**	Helioscopinolides H	*E. calyptrata*	[40]
**21**	*ent*-11α-Hydroxyabieta-8(14),13(15)-dien-16,12α-olide	*E. ebracteolata*	[25]
		*E. sessiliflora*	[30]
		*E. fidjiana*	[33]
**22**	*ent*-12-Hydroxy-12[R]-abieta-8(14 ),13(15)-dien-16,12-olide	*E. sessiliflora*	[30]
**23**	7β,11β,12β-Trihydroxy-*ent*-abieta-8(14),13(15)-dien-16,12-olide	*E. fischeriana*	[31]
**24**	Langduin B	*E. fischeriana*	[32]
**25**	Helioscopinolide C	*E. helioscopia*	[39,41]
**26**	Helioscopinolides F	*E. calyptrata*	[40]
**27**	Helioscopinolide D	*E. calyptrate*	[42]
**28**	Helioscopinolide E	*E. calyptrate*	[42]
**29**	Helioscopinolides I	*E. calyptrata*	[40]
**30**	8α,14-Dihydro-7-oxo-jolkinolide E	*E. characias*	[36]
**31**	8α,14-Dihydro-7-oxohelioscopinolide A [caudicifolin]	*E. sessiliflora*	[30]
		*E. characias*	[36]
		*E. semiperfoliata*	[38]
**32**	3,4,18β-Cyclopropa-8β-hydroxy-14-oxo-*ent*-abiet-13,15-en-16,12-olide	*E. retusa*	[43]
**33**	3,4,18β-Cyclopropa-14-oxo-*ent*-abieta-8,9,13,15-dien-16,12-olide	*E. retusa*	[43]
**34**	3,4,18β-Cyclopropa-14-oxo-*ent*-abieta-7,13,15-dien-16,12-olide	*E. retusa*	[43]
**35**	3,4,18β-Cyclopropa-7-hydroxy-14-oxo-*ent*-abieta-8,9,13,15-dien-16,12-olide	*E. retusa*	[43]
**36**	3,4,18β-Cyclopropa-14-oxo-*ent*-abiet-7-en-16,12-olide	*E. retusa*	[43]
**37**	*ent*-16-Hydroxy-13[R]-pimar-8(14) -ene-3,15-dione	*E. fidjiana*	[33]
**38**	*ent*-l2α,16-Dihydroxy-13[R]-pimar-8(14) -ene-3,15-dione	*E. fidjiana*	[33]
**39**	13β-Hydroxy-*ent*-abiet-8(14)-en-7-one	*E. fischeriana*	[31]
**40**	Methyl 8β,11β-dihydroxy-12-oxo-*ent*-abieta-13, 15(17)-dien-16-oate	*E. portulacoides*	[44]
**41**	11,16-Epoxy-*ent*-abieta-8,11,15-triene-13,14-dione	*E. guyoniana*	[34]
**42**	11-Hydroxy-*ent*-abieta-8,11,13-trien-15-one	*E. guyoniana*	[34]

**Figure 1:**
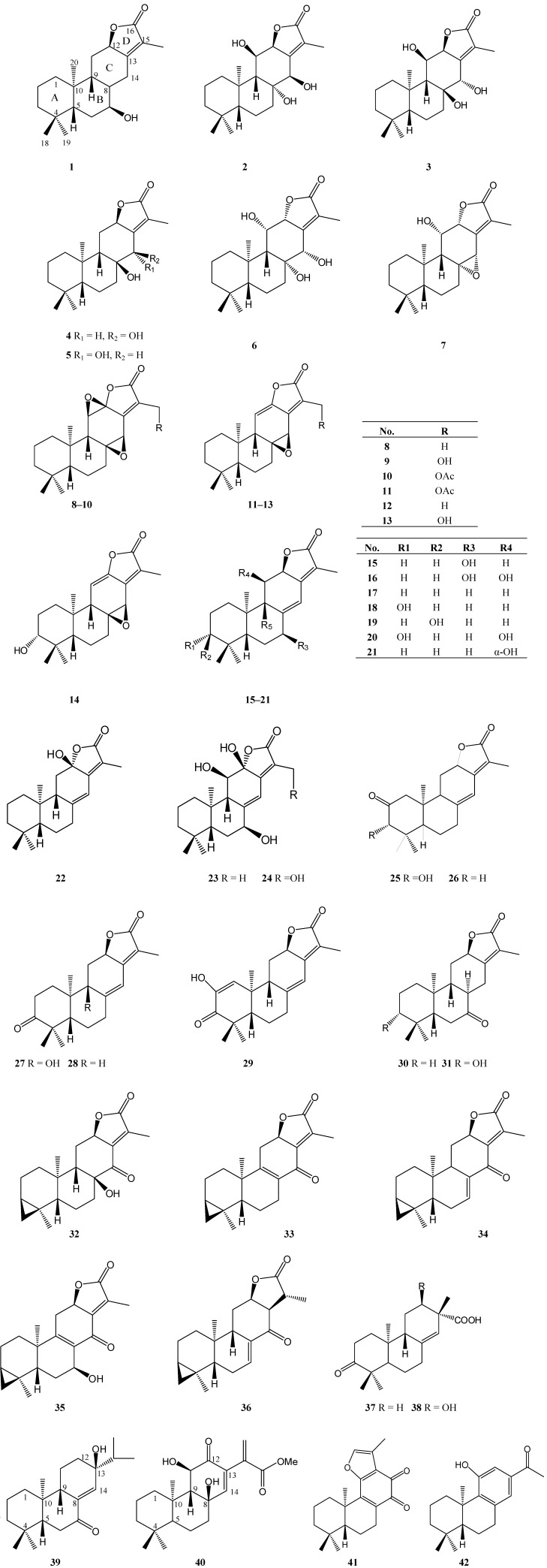
Abietane diterpenoids isolated from *Euphorbia* species.

## 3. Ingenane Derivates Isolated from *Euphorbia* Species (Table 2)

Ingenane diterpenoids have a very unique structural feature: they all have a same 5/7/7/3-tetracyclic ring system and a ketone bridge between C-8 and C-10. There is a double bond between C-1 and C-2 in ring A, and another double bond between C-6 and C-7 in ring B. A β-hydroxyl group is linked to C-4, so ring A/B must be *trans*-joined. Besides, ring D is a cyclopropane ring. Some positions at C-3, C-5, C-13, C-17 and C-20 may be linked to oxygen-substituted residues, such as hydroxyl, acetyl ester, long-chain alkyl ester, benzoyl ester groups, and so forth. This type of diterpenoids have been widely reported in many *Euphorbia* species. Some researchers have shown that these diterpenoids have antinematodal and termiticidal activity [45,46]. There were also reports about toxicity such as tumor promoting and proinflammatory activity [20,47,48]. Studies on the relationships between structure and irritant activity indicate that presence of a hydroxyl on C-20 is crucial for stimulatory properties. Introduction of an acetyl group in the 20-position results in a lower irritancy [49]. Some 20-deoxyingenol diterpenes induced cell cleavage arrest, but this activity became weak when C-16 had an acyl residue [50]. Acetylation in the 5-position resulted into a considerable depression of irritancy [49]. The skin tumor promoting and irritant activities of the ingenol-3-esters depend on the length of the aliphatic chain in their ester moiety [51]. In addition, the presence of one free hydroxy group at C-3 or C-5 may play an important role in the antinematodal activity [45].

**Table 2:** Ingenane diterpenoids isolated from *Euphorbia* species.

No	Name	Species	Ref
**43**	Ingenol	*E.kansui*	[45]
		*E. paralias*	[48]
**44**	13-*O*-Dodecanoylingenol	*E. kansui*	[45]
**45**	17-[(2Z,4E,6Z)-Deca-2,4,6-trienoyloxy] [ingenol]	*E. cauducifolia.*	[21]
**46**	20-Eicosanoate	*E. iberica*	[52]
**47**	3,5,20-*O*-Triacetylingenol	*E. kansui*	[45]
**48**	17-Hydroxyingenol tetraacetate	*E. kamerunica*	[53]
**49**	5,20-*O*-Diacetyl-3-*O*-(2″,3″-dimethylbutanoyl)-13-*O*-dodecanoylingenol	*E. kansui*	[45]
**50**	20-Tetradecanoate-ingenol-3,5-diacetate	*E. broteri*	[54]
**51**	17-*O*-Acetyl-3-*O*-[(Z)-2-methyl-2-butenoyl]-20-deoxy-17-hydroxy-ingenol	*E. trigona*	[55]
**52**	20-*O*-Acetyl-3-*O*-[(Z)-2-methyl-2-butenoyl]ingenol	*E. trigona*	[55]
**53**	5,17,20-*O*-Triacetyl-3-*O*-[(Z)-2-methyl-2-butenoyl]-17-hydroxyingenol	*E. trigona*	[55]
**54**	3-*O*-(2,3-Dimethylbutanoyl)-13-*O*-dodecanoylingenol	*E. kansui*	[45]
		*E. cyparissias*	[46]
**55**	3-*O*-(2,3-Dimethylbutanoyl)-13-*O*-decanoylingenol	*E. kansui*	[45]
		*E. cyparissias*	[46]
**56**	3,20-*O*-Diacetylingenol 5-*O*-(2'E,4'Z)-tetradecadienoate	*E. petiolata*	[56]
**57**	5,20-*O*-Diacetylingenol 3-*O*-(2'E,4'Z)-tetradecadienoa	*E. petiolata*	[56]
**58**	Ingenol-3-*O*-(2'E,4'Z)-tetradecadieno	*E. petiolata*	[56]
**59**	5,20-*O*-Isopropy1ideny1ingero1 3-*O*-(2'Z,4'Z)-tetradecadienoate	*E. petiolata*	[56]
**60**	20-*O*-Acetylingenol-3-*O*-(2"E,4"Z)-decadienoate	*E. petiolata*	[57]
**61**	20-Acetyl-ingenol-3-decadienoate	*E. broteri*	[54]
**62**	3-Tetradecanoate-ingenol-5,20-diacetate	*E. broteri*	[54]
**63**	5-Tetradecanoate-ingenol-3,20-diacetate	*E. broteri*	[54]
**64**	17-Benzoyloxy-3-*O*-(2,3-dimethylbutanoyl)-20-deoxyingenol	*E. esula*	[58]
**65**	17-Benzoyloxy-3-*O*-(2,3-dimethylbutanoyl)-13-(2,3-dimethylbutanoyloxy)-20-deoxyingenol	*E. esula*	[58]
**66**	17-Benzoyloxy-3-*O*-(2,3-dimethylbutanoyl)-13-(2,3-dimethylbutanoyloxy) ingenol	*E. esula*	[58]
**67**	13,17-Dibenzoyloxy-3-*O*-(2,3-dimethylbutanoyl)ingenol	*E. esula*	[58]
**68**	13,17-Dibenzoyloxy-3-*O*-(2,3-dimethylbutanoyl)-20-deoxyingenol	*E. esula*	[58]
**69**	3-*O*-(2,3-dimethylbutanoyl)-13-octanoyloxyingenol	*E. esula*	[58]
**70**	17-Benzoyloxy-3-*O*-(2,3-dimethylbutanoyl)-13-octanoyloxyingenol	*E. esula*	[58]
**71**	17-Benzoyloxy-20-*O*-(2,3-dimethylbutanoyl)-13-(2,3-dimethylbutanoyloxy)ingenol	*E. esula*	[58]
**72**	17-Benzoyloxy-13-octanoyloxyingenol	*E. esula*	[58]
**73**	20-*O*-Benzoyl-17-benzoyloxy-13-octanoyloxyingenol	*E. esula*	[58]
**74**	17-Benzoyloxy-20-*O*-(2,3-dimethylbutanoyl)-13-octanoyloxyingenol	*E. esula*	[58]
**75**	3-*O*-Benzoyl-17-benzoyloxy-13-(2,3-dimethylbutanoyloxy)ingenol	*E. esula*	[58]
**76**	3-*O*-Benzoyl-13,17-dibenzoyloxyingenol	*E. esula*	[58]
**77**	3-*O*-Benzoyl-13-octanoyloxyingenol	*E. esula*	[58]
**78**	3-*O*-Benzoyl-17-benzoyloxy-13-octanoyloxyingenol	*E. esula*	[58]
**79**	3-*O*-Benzoyl-17-benzoyloxy-13-octanoyloxy-20-deoxyingenol	*E. esula*	[58]
**80**	Ingenol-3-angelate-5,20-diacetate	*E. canariensis*	[59]
		*E. acrurensis*	[60]
**81**	5-Deoxyingenol-3-angelate-20-acetate	*E. canariensis*	[59]
**82**	17-Acetoxyingenol-5,20-diacetate-3-angelate	*E. kamerunica*	[53]
**83**	Ingenol-3-angelate	*E. canariensis*	[59]
**84**	17-Hydroxyingenol-3-angelate-17-benzoate	*E. canariensis*	[59]
**85**	17-Hydroxyingenol-3-angelate-20-acetate-17-benzoate	*E. canariensis*	[59]
**86**	17-Acetoxyingenol-20-acetate-3-angelate	*E. canariensis*	[59]
**87**	17-Hydroxyingenol 17-benzoate 20-angelate	*E. canariensis*	[59]
**88**	3-*O*-Angeloyl-17-[(2Z,4E,6Z)-deca-2,4,6-trienoyloxy]ingenol	*E. cauducifolia*	[21]
**89**	17-Acetyloxy-3-*O*-angeloyl-ingenol	*E. cauducifolia*	[21]
**90**	3-*O*-Angeloyl-17-(benzoyloxy)ingenol	*E. cauducifolia*	[21]
**91**	20-*O*-Acetyl-3-*O*-angeloyl-17-hydroxyingenol	*E. cauducifolia*	[21]
**92**	20-*O*-Acetyl-3-*O*-angeloyl-17-(benzoyloxy)ingenol	*E. cauducifolia*	[21]
**93**	3-*O*-Acetyl-20-*O*-angeloyl-17-hydroxyingenol	*E. cauducifolia*	[21]

**Figure 2:**
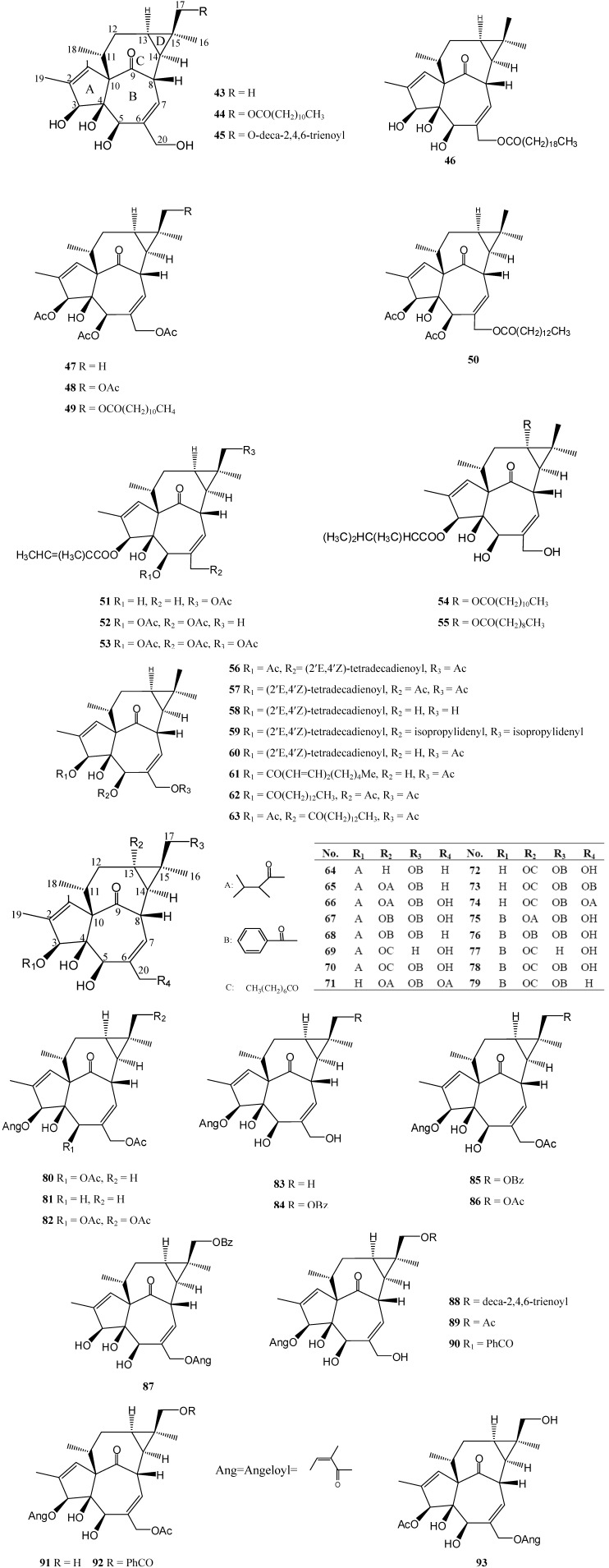
Ingenane Diterpenoids Isolated from *Euphorbia* Species.

## 4. Tigliane Derivates Isolated from *Euphorbia* Species (Table 3)

The tigliane diterpenoids in *Euphorbia* have a 5/7/6/3-tetracyclic ring system. Rings A/B are usually in *trans*-integrated configuration, as in compounds **94**–**98** and **100**–**120**. Only a few tigliane diterpenoids, such as **99** and **121**, are in *cis*-configuration. Rings B/C are joined in *trans*-configuration and Rings B/C in *cis*-configuration. Most tigliane diterpenoids have polyhydroxy groups located on C-4, C-9, C-13 and C-20. C-3 forms a carbonyl group. C1,2 and C6,7 form double bonds, respectively. Like the abietane and the ingenane diterpenoids, the hydroxyl groups of tigliane diterpenoids are easily esterified, as in compounds **98**–**103**. This type of macrocyclic deterpene, which is widespread in the seeds, roots, latex and stem of *Euphorbia* genus, is the main toxic constituent causing irritant, proinflammatory and tumor promoting activity [18,61,62]. When the C12-OH and C13-OH were esterified as a bis-ester, the tumor promoting activity was reinforced at the same time. For example, 12-*O*-tetradecanoylphorbol 12-acetate (TPA) is well-known as a tumor promotor. The diterpene ester with a saturated aliphatic long chain acyl group exhibited high irritant activity and high tumor promoting activity, and the highly unsaturated analogue exhibits high irritant activity, but very weak tumor promoting activity, suggesting that the irritant activity but not the tumour promoting activity of these diterpenoids is related to the degree of unsaturation of the aliphatic long chain [63]. The absence of a C20-OH is known to be important for the irritant and tumor promoting activities of phorbol esters [64]. Introduction of an acetyl group in the 20-position gives rise to a lower irritancy [65]. Compounds **122** and **123** belong to the daphnane diterpene group, which may be derived from the tigliane diterpenoids by cleavage of ring D and isopropenyl linked on C-13.

**Table 3:** Tigliane diterpenoids isolated from *Euphorbia* species.

No.	Name	Species	Ref
**94**	13-Acetoxy-12-deoxyphorbol [Prostratin]	*E. fischeriana*	[66]
**95**	20-Hydroxy-12-deoxyphorbol 13-(*cis*-9,10-methylene)-undecanoate	*E. poisonii*	[14]
**96**	20-Hydroxy-12-deoxyphorbol angelate	*E. poisonii*	[14]
**97**	12-Deoxyphorbaldehyde-l3-acetate	*E. fischeriana*	[31]
**98**	12-Deoxyphorbaldehyde-13-hexadecacetate	*E. fischeriana*	[31]
**99**	4,12-Dideoxy(4α)phorbol-13-hexadecanoate	*E. guyoniana*	[67]
**100**	12*-O-*(*2Z,4E*-Octadienoyl)-4-deoxyphorbol-13,20-diacetate	*E. broteri*	[54]
**101**	4,12,20-Trideoxyphorbol-13-(2,3-dimethyl)butyrate	*E. pithyusa subsp*	[68]
**102**	12*-O-*(*2Z,4E*-octadienoyl)-phorbol-13,20-diacetate	*E. broteri*	[54]
**103**	12-Deoxyphorbol-13-(*9Z*)-octadecanoate-20-acetate	*E. fischeriana*	[31]
**104**	13*-O-*Acetyl-20*-O-*benzoyl-12-deoxyphorbol	*E. cornigera*	[69]
**105**	13*-O-*Acetyl-20*-O-*p-methoxybenzoyl-12-deoxyphorbol	*E. cornigera*	[69]
**106**	13*-O-*Acetyl-20*-O-*decanoyl-12-deoxyphorbol	*E. cornigera*	[69]
**107**	13*-O-*Butanoyl-20*-O-*decanoyl-12-deoxyphorbol	*E. cornigera*	[69]
**108**	13*-O-*Hexanoyl-20*-O-*decanoyl-12-deoxyphorbol	*E. cornigera*	[69]
**109**	13*-O-*Octanoyl-20*-O-*decanoyl-12-deoxyphorbol	*E. cornigera*	[69]
**110**	13,20-Didecanoylphorbol	*E. cornigera*	[69]
**111**	13*-O-*Dodecanoyl-20*-O-*decanoyl-12-deoxyphorbol	*E. cornigera*	[69]
**112**	13*-O-*Decanoyl-20*-O-*angelyl-12-deoxyphorbol	*E. cornigera*	[69]
**113**	13*-O-*Decanoyl-20*-O-*tiglyl-12-deoxyphorbol	*E. cornigera*	[69]
**114**	12-Deoxyphorbol 20-acetate 13-angelate	*E. poisonii*	[14]
**115**	12-Deoxyphorbol 20-acetate 13-phenylacetate	*E. poisonii*	[14]
**116**	4,20-Dideoxyphorbol 12,13-bis(isobutyrate)	*E. obtusifolia*	[70]
**117**	4-Deoxyphorbol 12,13-bis(isobutyrate)	*E. obtusifolia*	[70]
**118**	17-Acetoxy-4-deoxyphorbol 12,13-bis(isobutyrate)	*E. obtusifolia*	[70]
**119**	17-Acetoxy-4,20-dideoxyphorbol 12,13-bis(isobutyrate)	*E. obtusifolia*	[70]
**120**	4-Deoxyphorbol 12,13-bis(isobutyrate) 20-acetate	*E. obtusifolia*	[70]
**121**	4-*Epi*-4-Deoxyphorbol 12,13-bis(isobutyrate)	*E. obtusifolia*	[70]
**122**	20-(4-Hydroxy-3-methoxyphenylacetate)9,13,14-orthophenylacetate	*E. poisonii*	[14]
**123**	20-Hydroxyresiniferol 9,13,14-orthophenylacetate	*E. poisonii*	[14]

**Figure 3:**
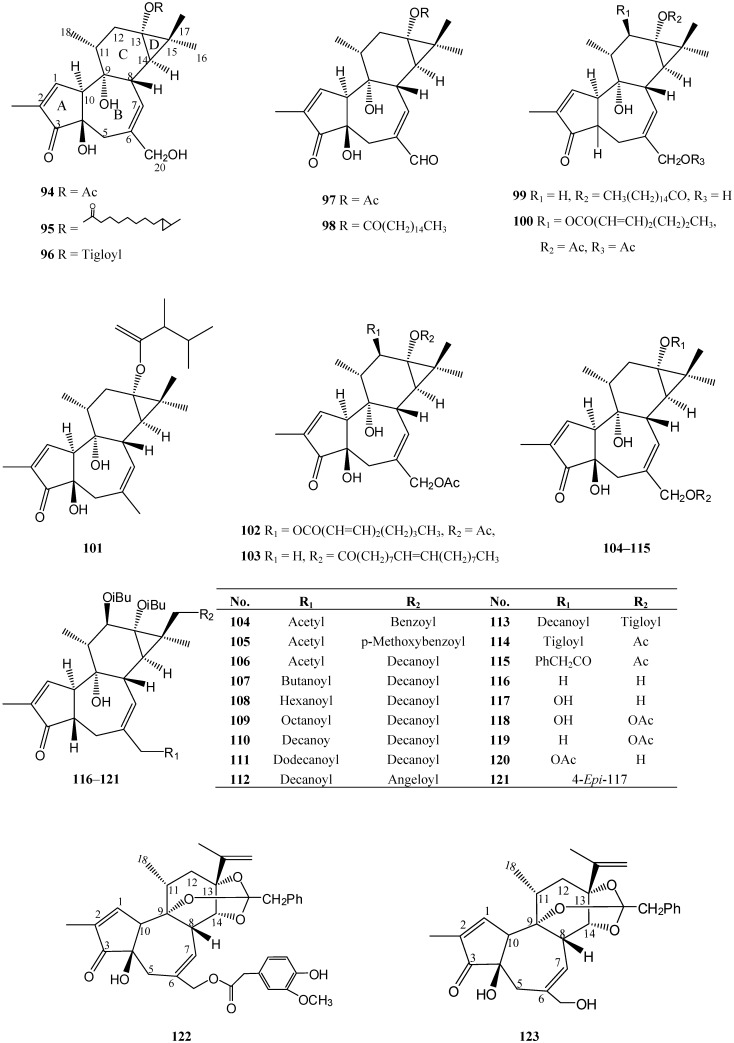
Tigliane diterpenoids isolated from *Euphorbia* species.

## 5. ^13^C-NMR Data of Diterpenes

Table 4 shows the ^13^C-NMR data of the diterpenoids **1–123**. All the ^13^C-NMR data were recorded in CDCl_3_. The structures and the carbon chemical shifts of the abietane diterpenoids are quite different from each other. Here we only discuss the most frequent abietane lactones **1**–**35**. Four carbons (C-12, C-13, C-15 and C-16) of the lactone ring are the main feature, and their chemical shifts are around δ_C_ 78.5–80.0, 148.4–165.0, 117.0–132.8 and 167.0–178.0, respectively. 

**Table 4:** ^13^C-NMR data (in CDCl_3_) of diterpenes from *Euphorbia* species.

Carbon	Compound / δ_C_ (in ppm)													
	**1**	**2**	**3**	**4**	**5**	**6**	**7**	**8**	**9**	**10**	**11**	**12**	**13**	**14**
1	38.0	40.9	38.3	43.2	40.5	41.9	41.6	41.4	41.4	41.3	40.0	41.4	39.3	37.6
2	18.4	18.0	17.3	20.1	18.7	17.4	17.1	18.5	18.5	18.5	18.4	18.4	18.4	27.0
3	42.0	40.9	41.3	43.2	43.1	39.9	38.6	40.0	39.2	39.0	41.5	39.8	41.5	78.2
4	32.7	32.3	32.4	34.2	34.1	37.8	37.6	33.3	33.5	33.5	33.5	33.4	33.5	39.2
5	46.8	54.7	55.3	56.6	57.0	55.6	55.4	53.2	53.6	53.5	53.5	53.4	53.5	52.9
6	30.2	19.8	16.7	22.0	19.2	18.0	17.7	21.2	21.0	20.9	20.8	20.8	20.9	20.5
7	68.9	39.9	34.9	43.1	36.3	35.5	35.4	36.5	36.6	35.7	33.8	34.0	34.0	33.9
8	35.1	75	74.4	75.7	77.6	75.0	74.8	60.8	66.9	67.4	61.3	61.1	61.3	61.0
9	42.5	62.6	56.3	58.0	47.4	72.0	72.1	66.6	47.0	47.8	51.9	51.7	51.8	51.6
10	38.0	36.9	37.2	40.3	39.0	40.5	40.5	48.2	39.1	39.3	41.6	41.3	41.4	41.1
11	27.5	67.3	65.0	29.9	29.0	57.2	65.4	61.3	61.6	61.9	107.6	104.1	106.4	103.4
12	78.5	79.0	79.7	79.0	79.9	79.7	79.8	85.4	85.5	85.3	149.5	147.4	147.3	147.6
13	163.4	157.5	160.3	165.3	166.5	161.2	160.3	148.4	150.8	154.5	147.2	144.9	146.5	144.8
14	26.7	71.9	71.8	73.5	74.3	65.2	57.4	55.6	53.6	55.3	54.3	54.4	54.4	54.3
15	120.5	124.2	125.9	123.2	125.5	125.0	126.0	130.1	150.8	128.3	122.3	125.1	127.4	125.4
16	175.4	175.4	176.2	178.0	177.8	175.4	175.3	169.8	168.2	167.4	170.5	170.6	169.2	170.4
17	8.4	6.7	7.9	8.2	9.4	9.2	9.0	8.6	56.5	54.9	55.4	8.6	56.3	8.6
18	33.1	33.0	32.4	34.6	34.1	16.7	16.4	33.2	33.5	33.5	33.4	33.4	33.5	28.3
19	21.6	20.8	20.6	22.4	22.4	21.8	21.3	22.1	21.9	21.9	21.9	21.9	21.9	15.5
20	12.6	16.8	15.7	18.1	15.4	33.6	33.7	15.4	15.6	15.1	15.0	14.9	15.1	15.0
Carbon	Compound / δ_C_ (in ppm)													
	**15**	**16**	**17**	**18**	**19**	**20**	**21**	**22**	**23**	**24**	**25**	**26**	**27**	**28**
1	41.9	31.7	39.7	37.4	32.1	29.9	39.4	39.0	39.6	40.2	51.2	55.9*	30.5	37.4
2	19.0	18.7	19.1	27.5	25.7	27.3	19.0	18.6	18.8	18.9	209.4	209.4	34.2	34.4
3	39.5	41.6	41.9	78.5	75.6	78.3	41.7	41.7	41.7	41.8	82.4	54.0*	216.4	215.6
4	33.1	33.2	33.6	39.0	37.8	39.0	33.6	33.4	32.9	41.0	45.0	38.7	47.1	47.5
5	47.1	39.9	55.3	54.3	48.4	45.4	55.4	54.0	46.8	46.7	53.4	54.5	46.0	54.8
6	31.0	31.0	23.9	23.4	23.4	23.0	23.8	22.3	29.9	30.7	23.0	23.6	24.1	24.6
7	72.4	74.4	37.2	36.8	37.1	32.7	37.1	36.0	71.5	71.1	36.3	36.4	32.2	36.6
8	151.2	148.4	156.3	151.4	152.0	152.6	152.6	154.4	153.4	155.2	149.4	149.5	152.2	150.2
9	46.7	79.1	51.9	51.5	51.6	77.2	60.8	51.4	54.8	55.3	51.3	51.3	76.9	50.7
10	41.9	44.7	41.6	41.2	41.3	44.2	40.3	38.9	40.9	32.7	46.9	46.2	43.8	40.9
11	27.2	38.4	27.5	27.5	27.5	39.7	64.6	31.2	69.6	70.2	27.6	27.6	40.0	27.8
12	76.1	77.2	76.1	75.9	76.0	77.1	79.4	102.4	102.7	104.3	75.3	75.3	76.9	75.6
13	155.1	153.9	152.3	156.0	156.0	154.7	150.1	154.2	152.8	156.0	155.0	155.0	154.6	155.5
14	115.9	118.8	113.9	114.2	114.1	115.7	113.5	113.4	114.7	115.4	115.2	114.9	115.9	114.8
15	118.9	130.0	116.2	116.4	116.4	117.9	118.2	116.3	121.0	124.0	117.5	117.3	118.1	117.1
16	174.9	174.3	175.4	175.3	175.2	174.7	175.4	173.1	173.6	172.1	174.9	174.7	174.6	175.1
17	8.5	8.6	8.3	8.2	28.7	28.9	8.5	8.1	8.4	55.5	8.3	33.5	27.1	26.5
18	33.6	33.8	33.9	28.6	22.2	16.0	33.9	33.5	32.9	32.9	29.5	23.0	21.7	21.8
19	21.7	22.0	21.8	15.6	16.7	17.5	21.8	22.0	21.6	21.4	16.4	17.3	17.8	16.2
20	16.1	17.4	16.8	16.7	8.2	8.4	17.3	14.6	14.3	14.4	17.3	8.2	8.3	8.3
Carbon	Compound / δ_C_ (in ppm)													
	**29**	**30**	**31**	**32**	**33**	**34**	**35**	**36**	**37**	**38**	**39**	**40**	**41**	**42**
1	23.4*	37.9	28.2	33.6	30.4	31.0	29.8	31.4	37.5	37.4	38.9	39.3	36.6	36.1
2	145.2	18.2	26.8	18.8	19.3	19.1	19.2	19.2	34.7	34.6	18.4	18.31	19.0	19.2
3	200.2	41.4	78.1	19.1	18.5	19.9	18.4	19.9	216.5	216	41.8	41.5	41.3	41.3
4	44.0*	32.7	37.2	15.7	16.4	14.7	16.6	14.8	47.8	47.8	33.2	33.1	33.6	33.7
5	52.6	53.9	43.8	51.1	47.6	44.8	41.6	44.1	55.0	54.7	49.8	54.2	52.0	52.8
6	23.2	38.9	35.7	22.7	20.6	27.5	28.9	27.6	23.1	22.7	37.5	19.21	17.3	18.9
7	36.7	209.8	209.4	33.5	24.4	140.4	62.5	139.9	35.5	34.6	200.7	41.01	26.2	33.0
8	148.9	44.2	49.4	76.1	134.6	136.8	135.7	134.8	139.9	139.9	138.9	69.5	143.4	139.1
9	48.3	50.1	53.0	52.6	160.5	41.6	164.9	40.6	49.0	50.0	51.8	61.3	150.3	141.5
10	41.7*	37.4	39.1	36.6	38.9	34.3	39.8	35.2	38.1	37.7	35.9	37.8	39.3	39.8
11	27.6	28.1	23.8	27	34.2	27.2	33.9	27.7	18.6	26.3	18.6	71.7	152.0	154.6
12	75.4	77.5	77.0	79.5	78.8	77.9	78.4	76.7	31.0	69.8	29.7	197.5	125.4	112.8
13	154.9	160.7	160.4	153.3	150.6	151.1	149.9	52.9	46.7	52.1	71.8	136.9	176.4	135.0
14	116.0	24.0	38.5	196	185.7	187.5	187.0	196.2	123.7	121.9	139.5	151.4	184.8	123.0
15	117.7	121.9	122.0	131.8	131.1	132.5	132.8	40.0	214.1	215.1	37.8	137.3	120.2	198.0
16	174.9	174.8	174.8	172.9	172.8	173.5	173.3	178.2	64.8	65.2	17.6	–	144.2	26.4
17	27.0	8.4	8.4	9.4	9.8	10.0	9.9	16.2	23.8	17.5	16.2	128.8	8.5	33.7
18	2.0	13.1	27.6	21.5	22.3	20.5	22.2	20.4	25.8	25.6	32.6	33.8	33.5	22.2
19	19.4	33.5	14.9	23.9	23.2	24.5	23.2	24.7	22.3	22.3	21.2	21.9	21.9	19.6
20	8.3	21.0	13.1	16.9	16.8	11.5	15.7	12.4	14.7	14.3	14.1	17.8	20.4	–
Carbon	Compound / δ_C_ (in ppm)													
	**43**	**44**	**45**	**46**	**47**	**48**	**49**	**50**	**51**	**52**	**53**	**54**	**55**	**56**
1	130.0	129.0	131.7	130.0	132.2	131.8	130.5	132.2	132.1	132.2	131.6	131.4	131.4	132.1
2	138.8	139.3	136.3	138.8	133.2	133.6	136.0	133.4	135.8	135.8	136.0	136.2	136.2	133.3
3	80.5	80.3	80.2	80.6	82.2	82.0	81.7	82.1	82.9	82.7	81.6	82.5	82.5	82.1
4	84.3	84.0	74.7	84.4	85.8	85.7	85.7	86.0	85.0	84.9	85.8	84.5	84.4	86.0
5	75.3	75.2	75.0	73.8	74.8	74.6	74.8	75.0	77.3	74.8	74.8	76.7	76.7	75.0
7	127.4	126.2	128.4	128.3	131.9	131.0	129.7	131.8	123.1	129.5	130.9	127.2	127.3	128.2
8	44.0	43.2	42.9	44.1	43.6	43.1	42.9	43.7	43.0	43.6	43.2	42.6	42.6	43.7
9	207.8	206.5	205.2	208.0	205.4	204.6	204.7	205.4	206.0	206.2	204.7	205.9	205.9	205.6
10	72.4	72.6	72.0	72.6	71.9	71.8	71.8	72.1	72.0	72.1	72.0	71.8	71.8	72.1
11	39.8	38.6	37.7	39.6	38.6	38.6	37.9	38.7	38.7	38.5	38.6	37.4	37.4	38.6
12	30.8	35.0	35.2	31.0	31.1	30.8	35.0	31.1	30.8	31.2	30.7	35.0	35.0	31.2
13	23.1	68.8	29.1	23.9	23.1	23.2	69.1	23.1	24.0	24.0	24.1	69.0	69.0	23.2
14	22.9	28.2	29.4	23.2	22.9	24.0	28.0	23.3	23.7	23.3	23.2	28.2	28.2	23.0
15	24.0	30.2	30.1	22.7	24.4	27.7	30.7	24.4	27.4	28.5	27.6	30.3	30.3	24.3
16	28.5	22.5	24.7	28.5	28.4	24.2	22.2	28.5	24.3	23.1	24.2	22.4	22.5	28.4
17	15.4	16.7	66.8	15.5	15.5	65.7	16.8	15.4	65.6	15.5	65.7	16.7	16.7	15.5
18	17.3	18.4	18.2	17.4	17.0	16.6	18.0	17.1	16.7	17.3	16.6	18.2	18.2	17.1
19	15.5	15.4	15.5	15.4	15.4	15.4	15.3	15.6	15.6	15.6	15.4	15.4	15.5	15.4
20	67.2	66.8	62.3	66.4	65.8	65.7	65.7	65.9	21.9	66.8	65.7	67.1	67.2	65.9
Carbon	Compound / δ_C_ (in ppm)													
	**57**	**58**	**59**	**60**	**61**	**62**	**63**	**64**	**65**	**66**	**67**	**68**	**69**	**70**
1	132.2	132.0	132.2	132.2	132.2	132.0	132.3	132.1	131.5	131.0	131.2	131.8	131.5	131.1
2	133.4	135.9	135.9	136.1	136.1	–	133.5	136.2	136.3	136.8	136.8	136.4	136.2	136.7
3	82.5	82.7	81.8	82.8	82.5	82.4	82.4	82.0	82.7	82.1	82.2	82.9	82.6	82.0
4	86.0	84.8	84.1	85.0	85.1	–	85.9	85.2	84.9	84.8	84.9	85.0	84.5	84.8
5	78.5	76.7	74.0	74.9	75.0	75.0	75.1	76.7	77.0	76.0	76.5	77.4	76.9	76.0
6	135.8	139.4	136.6	136.1	136.2	–	135.5	138.4	138.2	140.2	140.1	138.3	139.6	140.1
7	128.4	128.2	128.3	129.3	129.4	132.0	131.7	122.8	122.0	125.6	126.1	122.2	127.3	125.7
8	43.7	43.5	43.6	43.9	43.8	43.7	43.7	42.8	42.6	42.7	43.0	42.9	42.7	42.6
9	205.7	207.0	207.6	206.2	206.1	–	205.3	206.7	205.1	205.2	204.9	205	205.8	205.1
10	72.2	72.0	72.3	72.1	72.3	–	72.1	71.5	71.9	71.9	71.9	72.0	71.9	71.7
11	38.5	38.4	37.6	38.8	38.6	38.6	38.7	38.5	38.1	37.8	38.0	38.4	37.5	37.8
12	31.1	31.2	31.2	31.2	31.3	31.2	31.2	30.5	35.6	35.6	35.3	35.3	34.4	35.0
13	23.2	23.3	23.5	23.3	23.4	23.1	23.1	23.8	68.3	68.3	69.3	69.3	69.0	68.5
14	23.0	23.0	23.0	23.1	23.3	23.3	23.3	23.4	28.7	28.5	28.6	29.0	28.3	28.4
15	24.3	24.0	24.0	24.0	24.0	–	24.4	27.5	33.9	34.0	34.5	34.4	30.3	34.0
16	28.4	28.5	28.5	28.5	28.5	28.5	28.5	24.2	18.7	18.5	18.7	18.7	22.5	18.5
17	15.6	15.5	15.6	15.5	15.5	15.4	15.4	66.2	65.5	65.5	65.6	65.7	16.7	65.5
18	17.1	17.3	17.5	17.3	17.3	17.1	17.1	16.4	18.0	18.1	18.0	17.9	18.2	17.9
19	15.4	15.5	15.5	15.6	15.5	15.6	15.6	15.3	15.5	15.4	15.6	15.6	15.4	15.5
20	66.1	67.1	64.3	66.8	66.7	65.9	65.6	21.6	21.7	66.6	67.0	21.8	67.2	66.7
Carbon	Compound / δ_C_ (in ppm)													
	**71**	**72**	**73**	**74**	**75**	**76**	**77**	**78**	**79**	**80**	**81**	**82**	**83**	**84**
1	128.6	128.6	128.4	128.8	131.4	131.5	132.0	131.5	132.0	132.0	132.0	131.5	132.1	131.6
2	139.7	139.8	139.7	139.6	136.8	136.8	136.0	136.8	136.0	136.0	136.0	133.7	135.7	136.2
3	80.4	80.2	80.1	80.4	83.0	83.0	83.3	83.0	83.6	81.8	85.7	81.5	82.5	82.2
4	84.3	84.3	84.3	84.3	84.9	85.1	84.6	84.9	85.1	85.8	80.5	85.7	84.7	84.9
5	73.8	75.2	74.0	73.8	77.3	76.3	76.9	77.2	77.2	74.9	43.7	74.6	77.1	76.6
6	137.7	141.2	137.5	137.8	139.8	140.2	139.0	139.8	138.0	133.0	132.0	135.9	139.0	139.7
7	126.8	125.2	125.5	126.7	126.4	126.0	128.0	126.4	122.0	132.0	129.0	130.8	128.6	127.2
8	43.2	43.3	43.3	43.3	42.9	43.0	42.7	42.9	42.8	43.6	44.2	43.1	43.5	43.2
9	204.9	205.4	205.3	204.8	205.0	205.1	206.0	204.9	205.0	206.0	208.0	204.6	206.6	205.9
10	72.9	72.7	72.8	72.7	72.2	72.0	72.0	72.0	72.1	72.0	75.0	71.9	72.0	72.0
11	38.8	38.8	38.8	38.9	38.0	38.2	37.6	38.1	38.5	38.6	37.0	38.5	38.3	38.4
12	35.9	35.4	35.6	35.4	35.9	35.3	35.2	35.4	35.4	31.1	31.5	30.6	31.1	30.9
13	68.1	68.4	68.3	68.3	69.4	69.3	69.0	68.6	68.6	23.1	23.7	23.1	23.3	24.3
14	28.7	28.7	28.6	28.7	28.6	28.6	28.3	28.6	28.8	22.9	23.2	23.9	23.0	23.5
15	33.8	33.9	33.9	33.9	34.0	34.4	30.3	34.1	34.1	24.3	23.6	27.5	24.0	27.7
16	18.8	18.7	18.6	18.7	18.9	18.6	22.5	18.7	18.7	28.4	28.6	24.1	28.5	24.6
17	65.4	65.7	65.5	65.5	65.5	65.6	16.7	65.5	65.6	15.4	15.5	65.6	15.5	66.2
18	18.5	18.2	18.2	18.2	18.4	18.1	18.4	18.1	18.1	17.0	18.2	16.5	17.3	16.9
19	15.2	15.4	15.3	15.3	15.6	15.6	15.6	15.6	15.6	15.5	15.6	15.3	15.5	15.6
20	65.7	66.6	66.4	65.7	67.1	66.9	67.4	67.1	21.8	65.9	68.5	65.6	67.5	67.2
Carbon	Compound / δ_C_ (in ppm)													
	**85**	**86**	**87**	**88**	**89**	**90**	**91**	**92**	**93**	**94**	**95**	**96**	**97**	**98**
1	131.6	131.7	131.3	131.3	131.7	131.7	131.7	131.7	131.7	160.6	161.4	161.3	160.4	160.5
2	136.2	136.4	136.8	136.8	136.3	136.3	136.7	136.3	136.3	132.9	132.7	132.7	133.5	133.5
3	82.3	82.5	84.7	84.7	84.2	84.4	83.6	83.9	84.1	209.2	209.3	209.4	208.3	208.4
4	84.9	84.9	74.2	74.2	74.2	74.5	74.3	74.4	74.1	73.8	73.8	73.8	72.8	72.8
5	74.7	74.9	74.0	74.0	74.5	74.2	74.3	74.3	75.0	38.7	38.6	38.5	34.4	34.6
6	136.4	136.3	136.8	136.8	136.7	136.5	136.7	136.3	136.6	140.4	139.8	140.0	142.9	142.9
7	128.2	128.7	128.5	128.5	128.3	128.4	128.0	128.1	128.4	130.4	130.6	130.4	158.1	158.2
8	43.2	43.3	42.1	42.1	42.9	42.9	42.4	42.8	42.9	39.1	39.2	39.1	41.4	41.5
9	205.5	205.5	207.2	207.2	206.0	205.7	205.0	205.4	205.2	76.0	76.0	76.2	77.1	77.1
10	72.0	72.1	71.7	71.7	71.9	71.9	71.7	71.8	71.9	56.2	55.8	55.7	55.8	55.8
11	38.5	38.5	37.2	37.2	37.7	37.6	37.6	37.6	37.7	36.6	36.3	36.3	36.5	36.5
12	30.9	30.9	34.9	34.9	35.1	35.1	35.2	35.3	35.2	32.3	32.0	31.9	31.7	31.8
13	24.3	24.3	29.4	29.4	29.4	29.3	29.5	29.8	29.2	63.8	63.2	63.2	63.0	63.0
14	23.5	23.5	29.1	29.1	29.6	29.4	29.8	29.6	29.7	32.8	32.6	32.8	32.0	32.1
15	27.7	27.5	29.7	29.7	30.2	29.8	30.0	30.0	30.0	22.5	26.6	22.9	22.9	22.7
16	24.5	24.4	24.8	24.8	24.8	24.9	24.7	24.9	24.7	23.2	23.1	23.6	23.1	23.2
17	66.1	65.6	66.2	66.2	66.3	66.3	62.5	66.3	62.2	15.3	15.4	15.4	15.3	15.3
18	16.9	17.0	18.5	18.5	18.6	18.7	18.8	18.2	18.4	18.8	18.6	18.6	18.5	18.6
19	15.6	15.6	16.1	16.1	15.3	16.0	16.0	15.9	15.5	9.9	10.1	10.1	10.1	10.1
20	66.5	66.7	62.2	62.2	62.4	62.3	65.5	66.3	66.4	67.9	68.3	68.2	193.8	193.8
Carbon	Compound / δ_C_ (in ppm)													
	**99**	**100**	**101**	**102**	**103**	**104**	**105**	**106**	**107**	**108**	**109**	**110**	**111**	
1	156.9	159.7	161.0	160.8	161.3	160.3	160.3	160.3	160.3	160.3	160.3	160.3	160.3	
2	143.0	137.3	138.3	135.7	132.9	136.3	136.3	136.2	136.2	136.3	136.3	136.2	136.3	
3	213.8	208.9	203.0	208.6	208.9	210.2	210.3	210.3	210.3	210.3	210.3	210.3	210.3	
4	50.1	42.6	44.4	73.6	73.7	44.5	44.5	44.5	44.5	44.6	44.5	44.6	44.6	
5	25.1	35.1	34.0	38.8	38.9	34.0	34.0	34.1	34.0	34.0	34.0	34.1	34.1	
6	136.3	136.5	136.2	132.9	135.2	139.6	139.6	139.7	139.7	139.6	139.6	139.6	139.7	
7	127.7	130.2	126.8	132.7	133.7	125.0	125.7	125.7	125.7	125.7	125.6	125.6	125.6	
8	41.0	42.3	41.9	39.4	39.5	42.2	42.2	42.2	42.2	42.2	42.2	42.2	42.2	
9	75.5	77.8	75.2	78.2	75.9	77.9	77.9	77.9	77.9	77.9	77.9	77.9	77.9	
10	47.1	54.1	53.9	56.2	55.8	54.3	54.3	54.3	54.4	54.3	54.3	54.3	54.3	
11	37.1	44.1	46.2	43.2	36.3	42.3	42.3	42.3	42.3	42.3	42.3	42.4	42.4	
12	30.5	76.1	31.8	76.1	31.9	56.7	56.7	54.8	56.8	56.7	56.7	56.7	52.7	
13	62.7	65.4	62.8	65.7	63.6	65.0	65.0	65.0	65.1	65.0	65.0	65.0	65.0	
14	33.1	35.4	32.0	36.1	32.4	35.9	35.8	35.9	36.0	35.9	35.9	36.0	36.0	
15	22.5	25.7	22.5	25.7	22.6	25.8	25.8	25.8	25.8	25.8	25.8	25.8	25.8	
16	23.7	23.8	15.2	23.8	23.2	23.9	23.9	23.9	24.0	23.9	24.0	23.9	23.9	
17	15.2	16.7	22.9	16.7	15.3	17.0	17.0	16.9	16.9	17.0	16.9	16.9	16.8	
18	15.9	15.1	19.0	14.4	18.6	15.1	15.1	15.1	15.2	15.1	15.2	15.2	15.0	
19	10.4	10.2	10.0	10.1	10.1	10.2	10.2	10.3	10.3	10.2	10.3	10.3	10.3	
20	69.5	68.9	25.2	69.4	69.4	62.2	66.4	67.4	68.2	66.9	66.7	66.5	65.9	
Carbon	Compound / δ_C_ (in ppm)													
	**112**	**113**	**114**	**115**	**116**	**117**	**118**	**119**	**120**	**121**	**122**	**123**		
1	160.3	160.2	161.5	161.4	160.2	159.8	160.0	159.4	159.8	156.2	158.2	158.3		
2	136.2	136.2	132.8	132.8	136.3	136.4	136.6	136.5	136.5	143.3	136.6	136.5		
3	210.3	210.3	209.1	209.0	210.2	209.7	209.7	210.1	209.6	213.3	208.4	209.0		
4	44.5	44.5	73.6	73.6	44.5	44.2	44.0	44.3	44.1	49.6	73.3	73.5		
5	34.1	34.1	39.0	39.0	34.0	29.6	29.0	33.6	30.0	25.1	40.0	39.8		
6	139.6	139.7	134.8	134.8	139.0	142.0	142.4	139.3	137.2	137.0	135.0	138.9		
7	125.7	125.6	134.1	133.2	125.8	126.5	125.3	125.0	130.3	126.5	130.8	130.8		
8	42.2	42.2	39.5	39.4	42.2	42.1	42.4	42.6	42.2	40.7	39.1	38.9		
9	77.9	77.9	76.0	75.9	77.9	77.8	–	77.5	77.8	78.1	81.1	81.2		
10	54.3	54.3	55.7	56.7	54.3	54.2	53.8	53.9	54.0	47.4	55.4	55.5		
11	42.4	42.4	36.4	36.3	42.3	42.4	42.4	42.5	42.3	43.2	33.0	33.1		
12	53.8	56.8	31.9	31.7	76.7	76.7	76.1	76.2	76.4	75.3	35.7	35.7		
13	65.0	65.1	63.1	63.9	65.0	65.0	65.2	65.3	64.8	64.8	84.4	84.5		
14	36.0	35.9	32.7	32.3	35.9	35.8	36.4	36.5	35.5	37.1	80.6	80.8		
15	25.8	25.8	22.9	23.0	25.8	25.9	30.0	29.8	25.8	25.3	146.5	146.4		
16	23.9	24.0	23.6	23.0	23.9	23.8	–	19.5	23.8	24.2	110.7	110.7		
17	17.0	16.9	15.4	15.3	16.9	16.9	63.3	63.5	16.8	16.5	18.8	18.8		
18	15.2	15.2	18.6	18.5	15.1	15.1	15.2	15.2	15.0	11.9	19.8	19.9		
19	10.3	10.3	10.1	10.1	10.2	10.2	10.3	10.3	10.3	10.5	10.2	10.2		
20	67.2	65.9	69.8	69.7	25.4	67.5	67.1	25.4	68.9	69.3	70.4	69.3		

(–) Data not observed; *interchangeable.

Affected by the α,β-unsaturated γ-lactone, the carbon chemical shifts of Me-17 is very low at δ_C_ 6.7–10.0, as shown in **1**–**7**, **12**, **14**–**18**, **21**–**23**, **25** and **30**–**34**. Thus, the assignment of the four methyl groups (C-17, C-18, C-19 and C-20) in compounds **19**–**20** and **26**–**29** [40,42] were doubtful. In their ^13^C-NMR spectra, chemical shifts around δ_C_ 8.0 should be assigned to C-17 instead of C-20, δ_C_ 27.0-34.0 should be assigned to C-18 instead of C-17, and δ_C_ 16.0–23.0 should be assigned to C-19 instead of C-18. Other positions, such as C-2, C-3, C-7, C-8, C-11 and C-14 are usually substituted by oxygen groups, whose carbon chemical shifts are around δ_C_ 65.0–78.0. Values close to δ_C_115.0 can be assigned to tertiary carbon (C-14) on double bond in **15**–**29**. Some carbons of the abietane diterpenoids (**27**, **28** and **30**–**36**) may be carbonylated when their chemical shifts are above δ_C_ 185.0.

The carbon chemical shifts do not show very characteristic features for ingenane skeleton type. Their characteristic carbon chemical shifts are observed around δ_C_ 127.0–140.0 and 204.0–208.0, which are assigned to four carbons (C-1, C-2, C-6 and C-7) on two double bonds and the bridged carbonyl (C-9). The carbon chemical shift around δ_C_ 71.8–72.8 is assigned to the quaternary carbon (C-10) near the bridged carbonyl. The carbon chemical shifts around δ_C_ 74.0–86.0 (assigned to C-3, C-4 and C-5 with oxygen substituted) are registered in the ^13^C-NMR spectra of these compounds. Besides, there is another carbon (C-20) usually oxygen substituted, but it show little lower values (δ_C_ 62.3–67.2), while its value is around δ_C_ 21.6–21.8 without oxygen substitution, as the compounds **64**, **65** and **68**. The carbon chemical shifts of **65**–**79** show C-13 and C-17 are registered δ_C_ 65.5–69.1 after acylation.

The structures of tigliane diterpenes can be confirmed by carbon chemical shifts around δ_C_ 203.0–210.3, 156.2–160.6, 132.7–138.3, 132.9–142.9 and 125.0–158.2 assigned to C-3, C-1, C-2, C-6 and C-7, respectively (except **99** and **121**). Values close to δ_C_ 73.0 and 77.0 are assigned to the carbons C-4 and C-9 substituted by hydroxyl groups. The carbon chemical shift of C-20 is at δ_C_ 193.8 when it is substituted by hydroxyl group (**97** and **98**), as well as the chemical shift of C-7 is obvious higher than other tigliane diterpenes because of conjugated effect. Compounds **99** and **121 **are rare A/B *cis*-integrated compounds, and the structure of these isomers can be confirmed from the data of ^13^C-NMR, for the chemical shifts of C-2 and C-3 are 4–7 ppm higher than that of the A/B *trans*-integrated ones.
